# Incidence of Hepatitis C Infection among Prisoners by Routine Laboratory Values during a 20-Year Period

**DOI:** 10.1371/journal.pone.0090560

**Published:** 2014-02-28

**Authors:** Andrés Marco, Carlos Gallego, Joan A. Caylà

**Affiliations:** 1 Health Services of Barcelona Men’s Penitentiary Centre, Barcelona, Department of Justice, Government of Catalonia, Barcelona, Spain; 2 Health Services of Quatre Camins Penitentiary Centre, La Roca del Vallés, Barcelona, Department of Justice, Government of Catalonia, Barcelona, Spain; 3 Epidemiology Service of the Public Health Agency of Barcelona, CIBER of Epidemiology and Public Health (CIBERESP), Barcelona, Spain; Duke University, United States of America

## Abstract

**Background:**

To estimate the incidence of Hepatitis C virus (HCV) and the predictive factors through repeated routine laboratory analyses.

**Methods:**

An observational cohort study was carried out in *Quatre Camins* Prison, Barcelona. The study included subjects with an initial negative HCV result and routine laboratory analyses containing HCV serology from 1992 to 2011. The incidence of infection was calculated for the study population and for sub-groups by 100 person-years of follow-up (100 py). The predictive factors were determined through Kaplan-Meier curves and a Cox regression. Hazard ratios (HR) and 95% confidence intervals (CI) were calculated.

**Results:**

A total of 2,377 prisoners were included with a median follow-up time of 1,540.9 days per patient. Among the total population, 117 HCV seroconversions were detected (incidence of 1.17/100 py). The incidence was higher between 1992 and 1995 (2.57/100 py), among cases with HIV co-infection (8.34/100 py) and among intravenous drug users (IDU) without methadone treatment (MT) during follow-up (6.66/100 py). The incidence rate of HCV seroconversion among cases with a history of IDU and current MT was 1.35/100 py, which is close to that of the total study population. The following variables had a positive predictive value for HCV infection: IDU (p<0.001; HR = 7,30; CI: 4.83–11.04), Spanish ethnicity (p = 0.009; HR = 2,03; CI: 1.93–3.44) and HIV infection (p = 0.015; HR = 1.97; CI: 1.14–3.39).

**Conclusion:**

The incidence of HCV infection among prisoners was higher during the first part of the study and among IDU during the entire study period. Preventative programs should be directed toward this sub-group of the prison population.

## Introduction

Hepatitis C virus (HCV) was identified and characterized in 1989 through the detection of non-hepatitis A or B genomes [1]. An estimated 150 million people have chronic HCV infection, though epidemiology and treatment of the disease have changed remarkably in recent years. The association between HCV and with intravenous drug use (IDU) has had a negative impact on HCV incidence, while advances in the safety of blood transfusions and prevention of nosocomial infection have improved the incidence [2].

An estimated 30–60% of new cases of HCV occur among the IDU in Europe [3], more than 80% of the new HCV cases in the U.S. occur among the IDU population [4], and more than 90% of the new cases in Australia [5]. In Spain, 1.6–2.6% of the population is infected with HCV, with disproportional representation from prisoners and the IDU population [6]. The presence of HCV infection among prisoners reached 48% in 1998 [7] and 22% in 2010 [8]. Changes in HCV prevalence in the general population and in the characteristics of the inmate population, such as a lower percentage of IDU, are responsible of the reduced incidence of HCV infection in prisons in recent years. Furthermore, some interventions, including methadone treatment and needles exchange programs, have also been instated in recent years.

The prevalence of HCV infection among the IDU population in Spain is 65–99% and is highest among individuals who are also co-infected with human immunodeficiency virus (HIV). Little data is available on the incidence of HCV since many individuals have an asymptomatic acute infection, making acute HCV infection underreported.

One way to improve the accuracy of HCV epidemiology is to quantify the incidence using the routine blood analyses over time. This allows for the measurement of HCV incidence and the impact of preventative measures. It would be difficult to perform this type of study on the IDU population because follow-up would be hindered by poor adherence. A closed setting, such as a prison, eliminates this limitation and provides repeated contact over time.

The objective of the present study is to utilize the prison setting and routine laboratory analysis to estimate the incidence of HCV infection and its predictive factors among prison inmates.

## Materials and Methods

We used an observational retrospective cohort study design and calculated the incidence of HCV seroconversion among inmates of Quatre Camins Penitentiary Center (QCPC) in La Roca del Valles province of Barcelona, Spain. We included all prison inmates with more than one laboratory analysis that incuded HCV serology completed between 01/01/1992 and 12/31/2012 and with an initial negative HCV result.

QCPC was opened in 1989, is located in the La Roca de Vallés and houses approximately 1,800 inmates. The inmates are admitted to QCPC to complete their entire sentence or are transferred from other prisons where they were temporarily until sentencing.

Routine HCV, hepatitis B virus (HBV) and HIV serology is offered to all prisoners at QCPC. Serology is repeated annually on all inmates with negative results or who present with risk factors. Current coverage of HCV serology is over 90%.

The following variables were collected from each inmates’ electronic medical record: age, birthplace (Spain vs foreign), race and ethnicity, history of IDU, methadone treatment (MT), hepatitis B surface antigen (HBsAg) and HIV co-infection. Patients undergoing MT were included only if they received continuous methadone from the date of negative HCV serology to the last date of follow-up. The study was closed on 12/31/2011, at which time all cases were identified as infected with HCV or not.

Incidence was calculated for the entire study population and specific sub-groups per 100 person-years of follow-up (100 py). The time between negative and positive HCV serology was calculated for cases who seroconverted. The time between the first and last HCV serology result was calculated for cases who did not seroconvert. Incidence was also calculated.

We documented whether each patient who had seroconverted had been released from prison on any occasion between the negative and positive HCV serology.

The statistical analysis was performed using SPSS-PC V16. Qualitative data was expressed as absolute numbers, percents and a median with standard deviation (SD). Kaplan-Meier curves were used to graph the time until seroconversion using the log-rank test. A multivariate analysis was performed to determine the predictive factors for seroconversion using a Cox regression on variables with p<0.15 on the bivariate level. The hazard ratio (HR) and 95% confidence intervals (CI) were also calculated.

The study was approved by the Justice Department of the Catalan Government.

## Results

A total of 2,377 inmates were included in the study; all were male and presented with negative HCV serology on their initial laboratory analysis. Two laboratory analyses were performed on 39% of the study population, three were performed on 18% of the population, and 4 or more were performed on 43%. The average age was 39.7±11 years, 53.7% were born in Spain and 59.9% were Caucasian. Seven percent had a history of IDU, 2.6% were positive for HBsAg and 1.9% were co-infected with HIV. Table 1 shows the demographic characteristics of the population stratified by presence or absence of HCV infection.

**Table 1:** Demographic characteristics of all prisoners.

Variable	Total	Infection	No infection	*p-*value
	n = 2377(%)	n = 117(%)	n = 2260(%)	
Age (mean in years)	39.7±11	39.3±9.6	39.7±11	0.69
Born in Spain	1277 (53.7)	99 (84.6)	1178 (52.1)	<0.001
Caucasian	1425 (59.9)	92 (78.6)	1333 (59.0)	<0.001
History of IDU	168 (7.1)	65 (55.6)	103 (4.6)	<0.001
IDU with MT	13 (5.5)	3 (2.6)	10 (0.4)	0.08
Positive HBsAg	62 (2.6)	5 (4.3)	57 (2.5)	0.19
HIV infection	46 (1.9)	19 (16.2)	27 (1.2)	<0.001

Quatre Camins Penitentiary Center, Barcelona, Spain (1992–2011).

IDU: intravenous drug use; MT: Methadone treatment; HBsAg: Hepatitis B surface antigen; HIV: human immunodeficiency virus.

**Figure 1:**
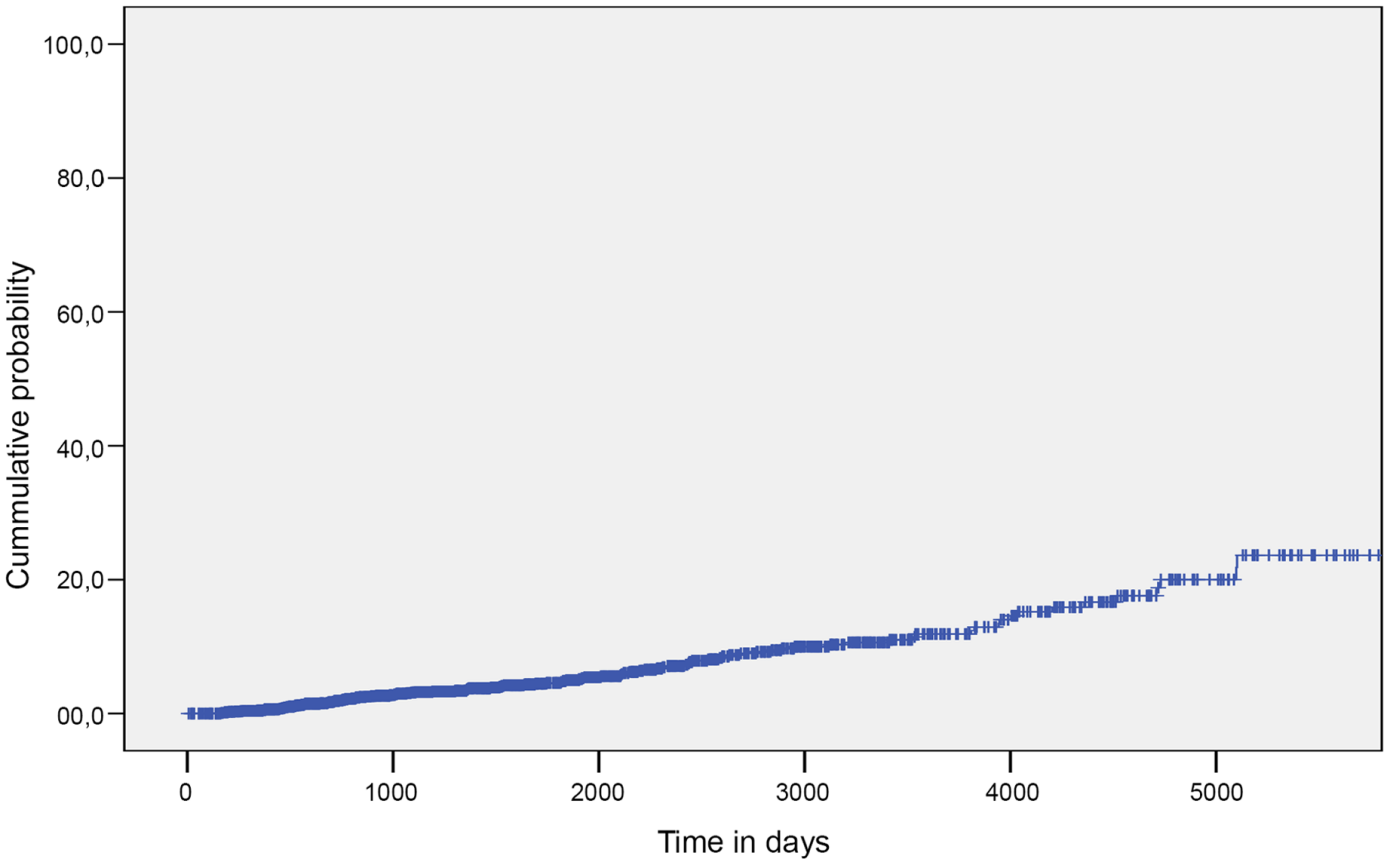
Probability of hepatitis C virus infection in 2,377 inmates. Quatre Camins Penitentiary Center, Barcelona, Spain (1992–2011).

**Figure 2:**
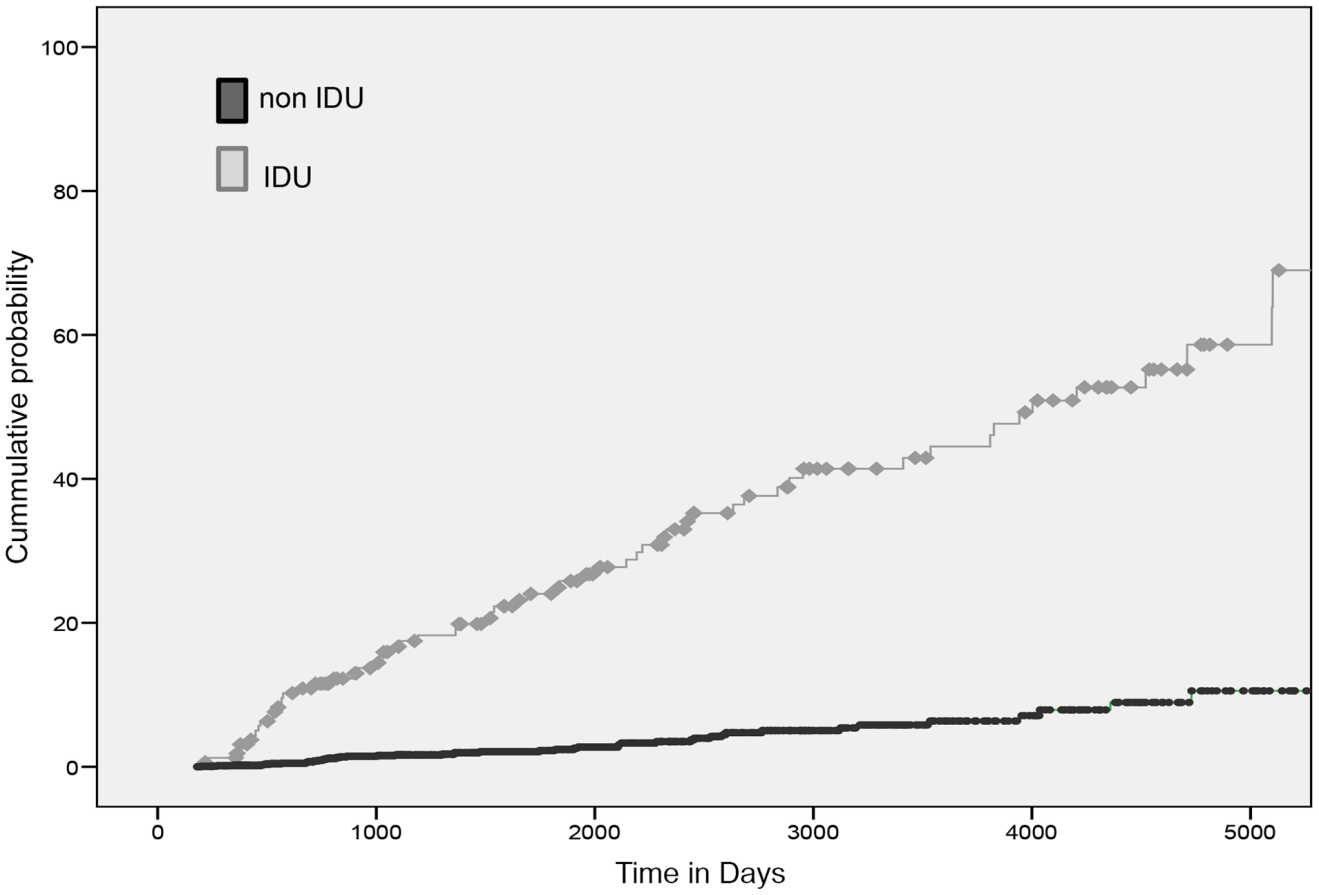
Probability of hepatitis C virus infection among intravenous drug users and non-intravenous drug users. Quatre Camins Penitentiary Center, Barcelona, Spain (1992–2011).

The total time of follow-up was 2,662,747 days, with an average of 1,540.9 days per patient. A total of 117 HCV seroconversions were detected (4.9% of the study population), which represent an incidence rate of 1.17/100 py (see Figure 1) and an accumulated probability of 15.4% (CI = 10.8–18.0) at 4,015 days (11 years). Among the subjects with no history of IDU, 51 HCV seroconversions (2.4%) were identified, correlating with an incidence rate of 0.58/100 py. Among the subjects with a history of IDU, 66 (38.7%) HCV seroconversions were identified, correlating with an incidence rate of 6.66/100 py. This also represents an accumulated probability of 18.2% (CI = 12.0–24.6) at 1,095 days (3 years) and 52% (CI = 37.4–58.6) at 4,015 days (11 years) (Figure 2).

A total of 29 HCV-infected inmates stayed within the prison during their follow-up time, corresponding to 17.3% inmates with a history of IDU and 44.6% of the inmates with a history of IDU and HCV infection. It was assumed that these individuals seroconverted within the prison. The rest of the study population had permission for release during the study period and thus we could not determine if HCV infection occurred within or outside the prison.

Among HCV-infected subjects, the average time until conversion was 6.3 years for those without a history of IDU and 5 years for patients with a history of IDU (p = 0.47).

**Figure 3:**
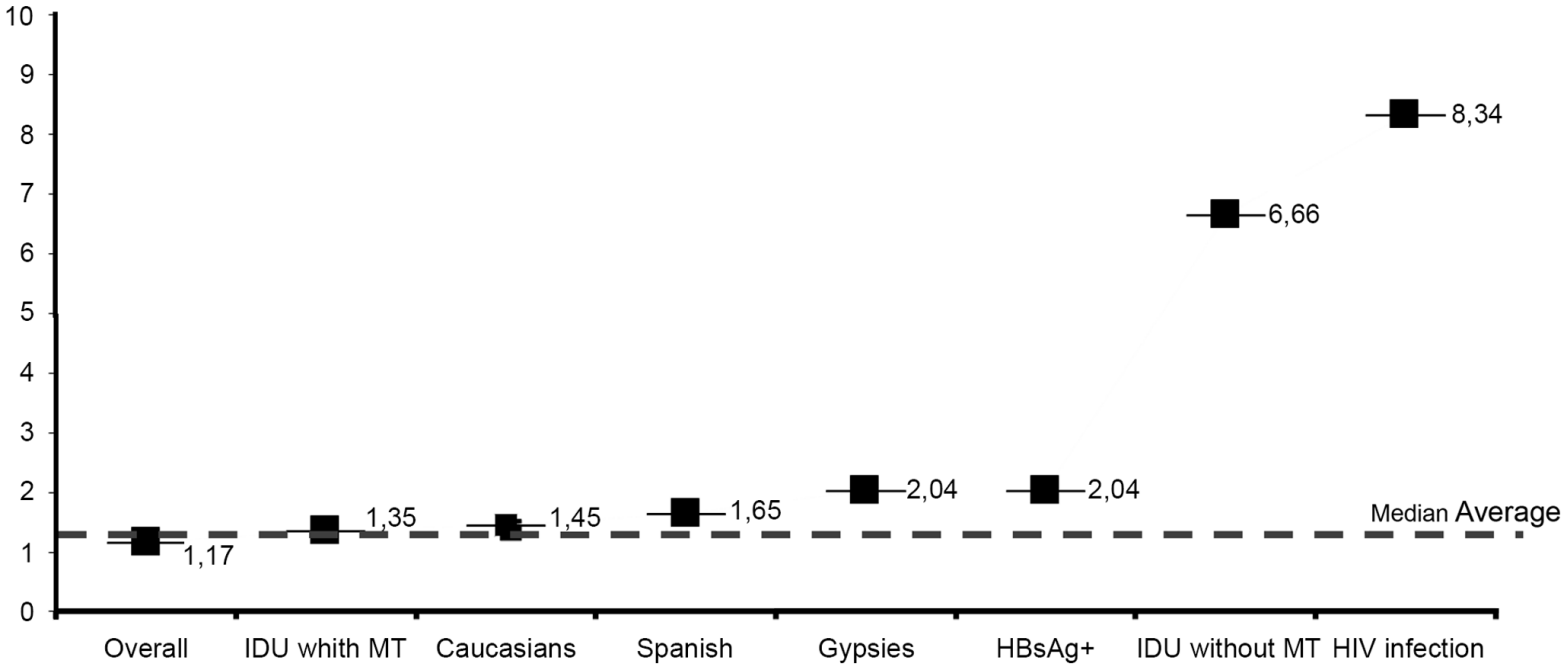
Incidence per 100 person-years of hepatitis C virus seroconversion. Quatre Camins Penitentiary Center, Barcelona, Spain (1992–2011).

The incidence of seroconversion was higher among cases with HIV co-infection (8.34/100 py) and among those with a history of IDU without MT during follow-up (6.66/100 py). The incidence rate of HCV seroconversion among cases with a history of IDU with current MT was 1.35/100 py, close to that of the total study population (1.17/100 py) (Figure 3).

**Figure 4:**
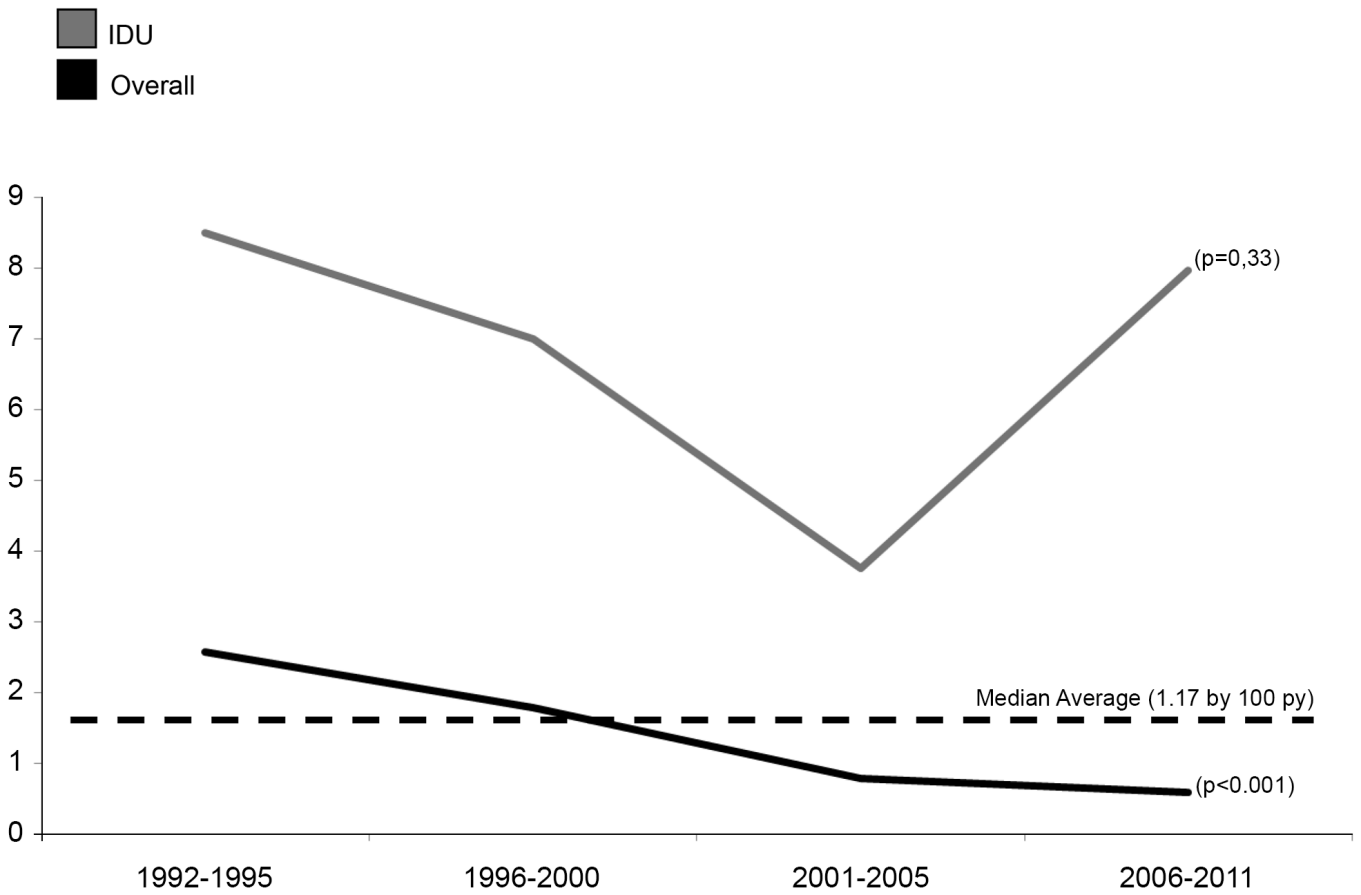
Incidence per 100 person-years of hepatitis C virus seroconversion among intravenous drug users compared to the overall study population by period in years. Quatre Camins Penitentiary Center, Barcelona, Spain (1992–2011).

A statistically significant reduction in incidence was observed over time; the incidence rate was 2.57/100 py from 1992–1995 and was 0.59/100 py from 2006–2011 (Figure 4). HCV seroconversion was reduced by 73% during 2006–2011, compared to 1993–1995. The incidence rate among cases with a history of IDU also decreased, but not with statistical significance (p = 0.33).

The bivariate and multivariate analyses of the predictors of HCV seroconversion are presented in Table 2. On the bivariate level, the following variables were associated with seroconversion: birthplace in Spain, Caucasian race, history of IDU, and HIV infection. MT was a protective factor against HCV seroconversion. On the multivariate level, the following variables were independently associated with seroconversion: birthplace in Spain, history of IDU, and HIV infection, while the association with race and MT were eliminated.

**Table 2:** Predictors of the incidence of hepatitis C virus infection in Quatre Camins Penitentiary Center, Barcelona Spain (1992–2011).

Variable	Bivariate analysis*		Multivariate analysis**	
	Infectionn (%)	p-value	p-value	Hazard Ratio(95% CI)
Age group (years)		0.91		
20–29	6 (1.5)			
30–39	68 (7.0)			
40–49	28 (4.8)			
≥50	15 (3.5)			
Born in Spain		<0.001	0.009	2.03 (1.19–3.44)
Yes	99 (7.8)			
No	18 (1.6)			
Race or ethnicity		0.002	0.27	0.85 (0.48–1.50)
Caucasian	92 (6.5)			
Gypsy	10 10.1)			
Maghreb	10 (1.8)			
Other	5 (1.7)			
Period (years)		<0.001	0.41	0.87 (0.63–1.20)
1992–1995	6 (1.5)			
1996–2000	68 (7.0)			
2001–2005	28 (4.8)			
2006–2011	15 (3.5)			
History of IDU		<0.001	<0.001	7.30 (4.83–11.04)
Yes	65 (38.7)			
No	52 (2.4)			
IDU with MT		0.08	0.91	1.07 (0.33–3.46)
Yes	3 (23,1)			
No	62 (40,0)			
HIV infection		<0.001	0.015	1.97 (1.14–3.39)
Yes	19 (41.3)			
No	98 (4.2)			
Positive HBsAg		0.21		
Yes	5 (8.1)			
No	99 (4.9)			
TOTAL	117 (4.9)			

Bivariate and multivariate analysis.

NS: Not statistically significant; CI: confidence interval; IDU: intravenous drug use; MT: Methadone treatment; HIV: human immunodeficiency virus; HBsAg: Hepatitis B surface antigen.

Long-rank test*. Cox regression**.

## Discussion

In recent years, the pattern of drug use in Spain has changed, resulting in a smaller proportion of IDU. This could explain the decreased incidence of HCV infection among the prison population. We consider the incidence rate of HCV of our study (1.17/100 py) to be high. Our incidence is much higher than that of a study regarding transplant patients (0.01/100 py) [9] and than that found in a meta-analysis of haemodialysis patients in developed countries (0.99/100 py) [10]. As expected, the incidence rate of HCV in our study was 11.5 times higher among IDU cases (6.66/100 py) than non-IDU cases. This rate, however, is lower than the incidence rate of the IDU prison population in other studies, which range from 12/100 py to 31.6/100 py [11], [12]. The rate of HCV among IDU cases in our study is also lower than the non-imprisoned IDU population in other studies, which range from 8.2/100 py to 46/100 py [13]–[19].

The difference in incidence rates may be due to the type of study and specific characteristics of QCPC, which has offered a methadone treatment program and needle exchange program. Additionally, other studies have been performed on active IDU cases, and we do not know if the IDU population in our study actively consumed during the study period. We would expect the incidence rate of HCV would be higher if the IDU population consumed during the study period. A study performed in Australia [20] reported that more than 50% of inmates with a history of IDU consumed while in prison, however other studies have reported a lower prevalence of intravenous drug consumption in prison [21], [22].

The QCPC has had a MT program for 18 years and currently treats 150 prisoners. Some studies have found that MT reduces the rate of HCV seroconversion [13], [23], [24], as well as other infections [13], [25], [26]. We found that the IDU population undergoing MT were 5 times less likely to seroconvert than IDU inmates with no MT (1.35/100 py per 6.66/100 py). MT was a protective factor in the bivariate analysis but was not statistically significant in the multivariate model. This could be because the small number of cases of IDU undergoing MT produced low statistical power.

The QCPC and other prisons in Spain also offer a needle exchange program [27], [28], which is a recommended strategy to limit intravenous transmission of infections [24], [29]. The average time until HCV seroconversion in our study was 5 years, compared to 3.3 and 3.4 years reported in other studies on non-imprisoned IDU cases [30], [31]. The incidence of HCV infection was also likely influenced by these preventative activities.

The incidence rate among inmates without a history of IDU in our study (0.58/100 py) was lower than that found in other studies, such as in Scotland (1.0/100 py) [8]. It is possible that transmission occurred more often through tattooing in these cases [13], [32], [33], however this has been disputed [21]. One study recently stated that HCV transmission via tattooing is highly unlikely when performed in the adequate setting, but more probable when it is performed in non-sterile conditions, such as in prisons, between friends, or without professional assistance [34]. Transmission through sexual contact, especially among the HIV-infected population [35], and through traumatic altercations [36], could both have also contributed.

In general, incarceration does not equate the absence of drug consumption. Published literature estimates that 60% of inmates with a history of IDU have used injected drugs in prison [37]. Furthermore, other studies report HCV seroconversion of HCV and other infections in prison [22], [38]–[41], mainly among the imprisoned IDU population. Twenty-nine prisoners with HCV seroconversion and a history of IDU did not leave the prison during our study, 44.6% of the total number of HCV infections among inmates with a history of IDU.

As seen in our study, the incidence rate of HCV infection has decreased during the last 20 years in Spain. The rate decreased from 6.8/100,000 py in 1997 to 1.46/100,000 py in 2011 [42] in Spain, and similar trends have been documented in the United States [43], [44] and other developed countries [45], [46]. Mathematic models predict that the long-term effects of chronic HCV infection will continue to increase in the future, despite this recent decline in incidence [43], [44], [46]. The incidence rate of HCV infection among prisoners with a history of IDU has not significantly decreased in Catalonia, where QCPC is located. This could be due to the significant proportion of inmates with a history of IDU who continue high-risk activities related to drug injection [47]. These high-risk activities have also been described in other geographical regions [16], [18], [45], [48] and among IDU patients [15], [16], [18] It has been suggested that better access to sterile needles is needed and target groups should include inmates who inject drugs [42].

There are a number of limitations in our study. We used a retrospective cohort study design and analyzed variables for which information was available. Thus the investigators could not choose the variables in a prospective manner. However, the study design also has the following advantages: it is economical, allows for the calculation of incidence rates and predictive variables through a multivariate analysis, and eliminates an outcome bias.

We acknowledge that data from only one prison is a limitation of our study which could influence the relevance of our results for other prisons. Nonetheless, the QCPC is similar to other Catalan prisons concerning population characteristics, availability of MT and needle exchange programs, and HCV testing (83.3% in September 2013). Population and health data is collected, analyzed and recorded on the Correctional Services of the Department of Justice for Spain [49], however, we cannot generalize our results to inmates in other countries because of differences in penitentiary policies and the socio-cultural characteristics.

Though our results only apply strictly to the study population, they can also be extrapolated to prison populations with a high proportion of inmates with a history of IDU in developed countries, especially in prisons with MT and needle exchange programs.

Finally, the incidence of HCV infection using this model of routine serology testing is simple, practical, and economical. We propose this to monitor incarcerated populations. Our study demonstrates that the incidence of HCV infection among the incarcerated population is declining in general, but to a lesser extent among inmates with a history of IDU. We therefore recommend the implementation of preventative programs directed at inmates with a history of IDU who continue to practice high-risk behaviour.
